# Transient bilateral striatal lesion associated with varicella infection

**DOI:** 10.1590/0100-3984.2016.0058

**Published:** 2017

**Authors:** Roberta Dantas Azevedo, Fabiano Reis

**Affiliations:** 1 Faculdade de Ciências Médicas da Universidade Estadual de Campinas (FCM-Unicamp), Campinas, SP, Brazil.

**Figure 1:**
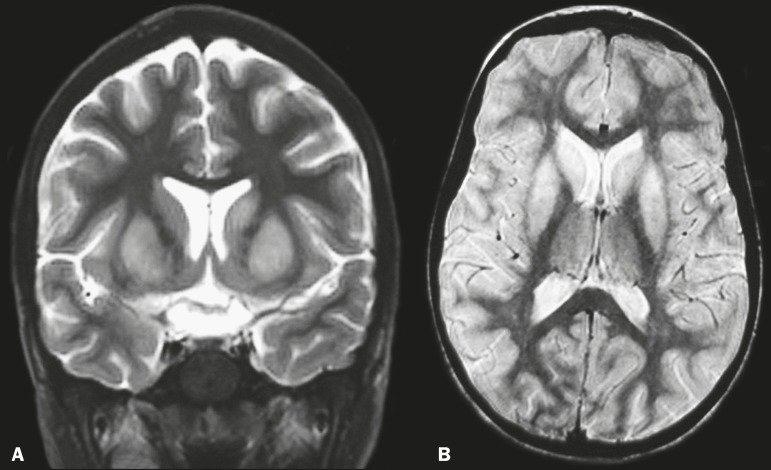
MRI of the brain showing hyperintense lesions in the caudate nuclei and putamen. A: Coronal T2-weighted sequence. B: Axial proton density-weighted sequence.

**Figure 2:**
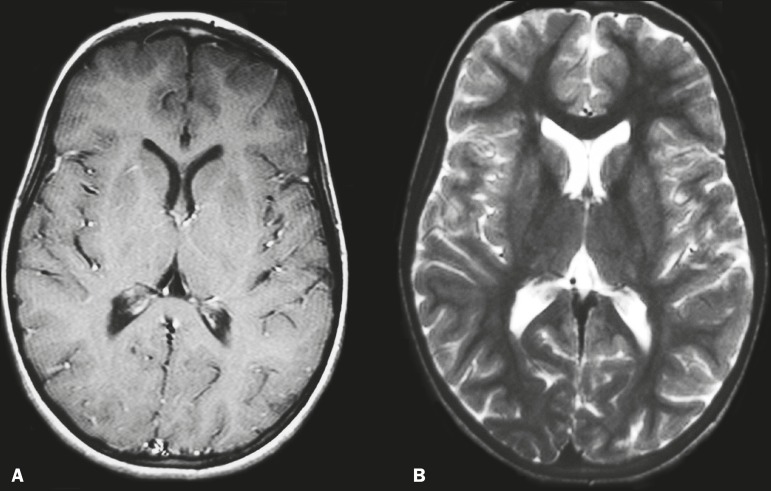
Follow-up MRI of the brain, three months after the first, showing regression of the lesions. A: Gadolinium contrast-enhanced axial T1-weighted sequence. B: Axial T2-weighted sequence.

Dear Editor,

A 5-year-old girl presented to our institution with an 8-day history of dermatological lesions typical of chickenpox, which had evolved to nausea, vomiting, and abdominal pain. During the observation period, she received symptomatic treatment (medication). Because she also experienced somnolence and apathy, she was hospitalized for further diagnostic investigation, evolving to a lack of fine motor coordination, difficulty in walking, tremor, dystonia, generalized tonic-clonic seizures, dysmetria, and decomposition of movement. Cerebrospinal fluid analysis revealed pleocytosis with a predominance of lymphocytes (12 leukocytes with 96% lymphocytes). A computed tomography scan of the head showed no abnormalities. Magnetic resonance imaging (MRI) showed hyperintense lesions in the caudate nuclei and putamen on T2-weighted and proton density-weighted sequences (Figure 1), without enhancement after contrast administration. The patient showed gradual improvement and was discharged after 6 days of hospitalization. She was referred to a pediatric neurology clinic. After three months of follow-up, her symptoms had completely disappeared and another MRI of the brain showed regression of the lesions (Figure 2).

Varicella-zoster virus causes chickenpox and is associated with a variety of complications. The most common noncutaneous site of involvement is the central nervous system. Complications include acute cerebellar ataxia, acute disseminated encephalomyelitis, and meningitis^(1)^. Bilateral striatal lesion (BSL) can also be seen during varicella infection^(2)^. BSL is a specific clinical syndrome with bilateral lesion of the basal ganglia, particularly the caudate nucleus and the putamen. The clinical presentation includes encephalopathy and irritability, together with variable pyramidal and extrapyramidal symptoms^(3)^. BSL can be found in conditions such as mitochondrial encephalopathy, hypoglycemia, and exogenous intoxication, although patients with those conditions show irreversible radiological changes and may have poor neurologic outcomes^(1)^.

The most common neurological complication of chickenpox is acute cerebellar ataxia^(4)^, which is a clinical syndrome characterized by a rapid onset of cerebellar dysfunction, manifesting primarily as gait disturbances and a loss of coordination^(5)^. Another disorder that affects the basal ganglia bilaterally is Epstein-Barr virus encephalitis. However, a diagnosis of Epstein-Barr virus encephalitis is made on the basis of the detection of the virus through polymerase chain reaction and positivity for immunoglobulin M in cerebrospinal fluid or blood samples^(6)^, neither of which were identified in our case.

Although Sydenham’s chorea is the most common poststreptococcal neuropsychiatric disorder, other disorders involving the basal ganglia after streptococcal infection, such as poststreptococcal dystonia, have been reported^(7)^. Because there is no specific diagnostic test, poststreptococcal dystonia is always a presumptive diagnosis^(7)^, although it becomes more likely if a temporal relationship is established between infection with group A beta-hemolytic streptococci and the onset of neurological symptoms. MRI findings observed in patients with Sydenham’s chorea can affect the central nervous system unilaterally.

Acute disseminated encephalomyelitis is another immune-mediated neurological complication that can occur after a viral infection or vaccination. It is an acute inflammatory demyelinating disease of the central nervous system, although lesions in the white matter and thalamus are seen on MRI^(8)^.

In the case presented here, the complete clinical recovery and the significant improvement of MRI findings favor the possibility of an immune-mediated striatal lesion as a complication of chickenpox.
